# Holistic view of patients with melanoma of the skin: how can health systems create value and achieve better clinical outcomes?

**DOI:** 10.3332/ecancer.2019.959

**Published:** 2019-08-27

**Authors:** Patrícia Redondo, Matilde Ribeiro, Machado Lopes, Marina Borges, Francisco Rocha Gonçalves

**Affiliations:** 1 Portuguese Oncology Institute of Porto, 4200-072 Porto, Portugal; 2 Management, Outcomes Research and Economics in Healthcare Group, Portuguese Oncology Institute of Porto, 4200-072 Porto, Portugal; 3 ENSP—Universidade Nova de Lisboa, Av. Padre Cruz, 1600-560 Lisboa, Portugal; 4 Luz Saúde—Rua Carlos Alberto da Mota Pinto, Edifício Amoreiras Square 17—9º, 1070-313 Lisboa, Portugal; 5 MEDCIDS/FMUP—Hospital de São João 9623, 4200-450 Porto, Portugal

**Keywords:** melanoma, patient care management, outcomes assessment, integrative oncology

## Abstract

Patients with skin cancer should be treated in healthcare units that ensure holistic and multidisciplinary approaches. Current healthcare units, especially those dedicated to cancer care, must evolve to integrated patient-centred systems.

The current review presents a holistic health services perspective towards managing patients with melanoma of the skin, based on a literature search. It includes a detailed discussion on how this could impact on the patient, his or her quality of life and on service providers.

Data from a multidisciplinary integrated practice unit, specialised in skin cancer, were also discussed, namely, for outcomes measurements, access to innovative treatments, value-based healthcare, patient centricity and use of integrated systems.

Epidemiology data, including disease determinants and risk factors, play an important role in defining measures, resources and management of these integrated cancer units. To optimise effective care and improve survival outcomes, integrated cancer clinics should comprise, in a patient-centred way, innovative treatments and technologies, along with continuous training and creation of multidisciplinary units of healthcare professionals.

Measurement of outcomes, such as clinical, quality of life and cost, is decisive in determining affordability and access to the best available state-of-the-art care. Besides, treatment of melanoma has significantly improved over recent years, but with increasing costs, which brings a challenging mission to guarantee access to treatment and quality care. Value-based healthcare allows the achievement of better health outcomes and higher quality services while reducing the costs associated with the full-care cycle.

Therefore, current healthcare systems should develop in line with health institutions’ organisation and culture, increasing adherence to best practices and create value.

## Introduction

One in every three diagnosed cancers is a skin cancer. There are 132,000 new cases of melanoma occurring worldwide every year, according to the World Health Organization [1]. Although non-melanoma skin cancers are more common, such as squamous cell carcinoma (SCC) and basal cell carcinoma (BCC), melanoma is one of the most severe types of skin cancer. Melanoma is originated from the melanocytes and is a complex cancer, with high genetic variability [2]. Apart from other rare types (e.g., mucosal surfaces, uveal tract and leptomeninges), cutaneous melanoma represents more or less 5% of all cutaneous malignancies but accounts for most related deaths [3]. In this review article, particular attention will be paid to melanoma of the skin.

Epidemiology data are an important foundation for management, evaluation and planning of integrated cancer units. Understanding the disease patterns and variations within populations provides better insights to define prevention, diagnostic and treatment actions for any disease [4]. Recently, a study predicting the incidence of melanoma in six Caucasian populations showed that cases will continue to increase, at least until 2022, with the ageing populations and high age-specific rates in the elderly [5]. These data are relevant to define long-term measures in healthcare systems in terms of resources and treatment access, in specific geographic regions. For instance, one major problem that might underestimate the actual melanoma burden is the lack of standardisation of registries among hospitals, laboratories and other primary care units [4].

In this review, the authors provide a holistic approach of the patient with melanoma of the skin and the factors impacting treatment and quality of life management, when followed-up in a multidisciplinary healthcare unit. Additionally, data from an integrated practice unit, specialised in skin cancer, are discussed, namely, for outcomes measurement, access to innovative treatments, value-based healthcare, patient centricity and integrated systems. A literature search was conducted in May 2018 to identify both original research and review articles, reporting the clinical burden and aetiology of melanoma. The most recent and representative publications were considered for this review if written in English and/or Portuguese and indexed in MEDLINE/PubMed, without date limits.

## Risk factors, biomarkers and prevention of melanoma of the skin

Melanoma of the skin is associated with multiple risk factors besides exposure to UV radiation (e.g., sun and tanning bed) and age, such as ethnicity (Caucasian population), history of blistering sunburns at young age, dysplastic nevi, family history, occupational chemicals’ exposure (e.g., arsenic), fair skin and hair (blonde or red hair) and immunosuppression [5]. A risk associated with male sex has been described, but postmenopausal women have shown different genetic patterns (polymorphisms), which increase the risk of developing melanoma by 1.9 times [6]. Nonetheless, melanoma of the skin can be prevented if the exposure to previous external factors, such as exposure to sun or tanning, is reduced. Several prevention campaigns and educational actions have been put in practice, but changes in behaviours are difficult to implement, even in younger adults [3]. Although also common in younger persons [3], it is predicted that the incidence of melanoma in this group might stabilise or decrease in forthcoming years [7].

In general, early detection of melanoma is a key factor for a good prognosis and survival of the patients. If diagnosed earlier in its localised stage (stage 0/I/II), the 5-year survival of cutaneous melanoma is approximately 98%–100% [3]. Standard procedures include biopsy followed by histopathological examination but advances of *in vivo* techniques with imaging/digital features allow a more precise, non-invasive detection [3]. In addition, skin self-assessment and total skin assessment (‘ABCDE’ criteria), by an experienced physician, are also recommended for screening skin cancer [8]. At an early stage, cutaneous melanoma can be removed by surgery with 80% recovery prognosis; however, survival decreases upon the development of metastases, which rapidly spread to other organs, especially to lungs [2].

While melanoma is one of the most complex tumours in terms of tumourigenic stability and molecular standardisation, this presents an opportunity to develop innovative treatments, such as immunotherapies and targeted therapies. One of the melanoma’s intricate features is the great number of cell pathways involved in its progression, as well as the cellular heterogeneity within aggressive melanoma. Consequently, subpopulations of cancer cells present stem cell properties, with multiple drug resistance (MDR) mechanisms (e.g., drug efflux pumps), while others show a high degree of plasticity, switching between different proliferation and malignancy stages [9].

During the last decade, an improved understanding of the physiopathology, clinical mechanisms and treatment of melanoma of the skin has occurred. Examples of the latter include multiple mechanisms in melanomagenesis, interference of the tumour microenvironment and regulation of the immune system, which led to the development of targeted and immune checkpoint therapies [10–12]. However, there is still a lot left to discover about biomarkers, genetic mutations and other factors involved in melanocytes’ growth, differentiation and progression in the skin [13, 14]. Currently, both targeted therapies and immunotherapies, focused on different genetic subtypes of cutaneous melanoma, are used in mono- and combined therapy and are preferred as first-line treatments [15], as described later.

## New treatment era for melanoma of the skin

Over the past 40 years, treatment of skin cancer, especially melanoma, has significantly improved. The first immunotherapy approaches for melanoma were the administration of Interferon-α2 (IFN α2) or interleukin (IL-2) in variable schedules, which presented high immunogenicity and toxicity effects, in addition to low response rates (overall complete response rate around 5%) [16, 17]. Since 2011, eight innovative targeted and immunotherapies were approved for metastatic or unresectable melanoma [17–20]. Prior to this treatment breakthrough, patients with advanced melanoma (stage IV) had a 5-year overall survival of 2.3% and a mean survival of 8–10 months [18].

Aiming at changing the lethality paradigm of patients with advanced melanoma, the first checkpoint inhibitor cytotoxic T-lymphocyte-associated protein-4, ipilimumab, was developed [16, 18–20]. Molecular targeted therapies were also developed to act on mutations of proteins from the mitogen-activated protein kinase pathway, such as B-Raf Proto-Oncogene, Serine/Threonine Kinase (BRAF) inhibitors (targeting BRAF V600E, R or K mutation, present in approximately 50% of melanoma tumours) and Mitogen Activated Protein Kinase (MAPK) Kinase (MEK) inhibitors [16, 18–20]. Additionally, programmed death protein 1 immune checkpoints have been approved, providing sustained long-term survival for these patients [16, 18–20]. More recently, an oncolytic viral therapy (talimogene laherparepvec), based on a genetically modified herpes simplex virus, was approved for locally advanced cutaneous and subcutaneous melanoma [16, 18–20].

Following those significant advances, combined therapies are being studied to overcome the already evident disadvantages of targeted and immunotherapeutic options in monotherapy (e.g., MDR) and to increase overall survival rate [16, 20, 21]. Comprising a potential synergistic strategy, combined therapies can be explored as the standard of care for optimal tailored treatment, based on individual features of melanoma patients (e.g., genetic biomarkers, comorbidities and clinical and biochemistry parameters) [16, 17]. Moreover, the use of these newer therapies in the adjuvant and neoadjuvant setting is being assessed to reduce the burden of surgical intervention and improve results for high-risk melanoma [10–12]. Further developments in pre-clinical and clinical research should focus on three main unmet needs: 1) new biomarkers to maximise cancer response to treatment, 2) evidence of improved long-term outcomes for patients and 3) possible increased toxicity with combination of therapies.

As a result of lengthening life expectancy, more population screening programmes and awareness as well as increasing exposure to UV radiation, the number of patients diagnosed with melanoma has risen [4, 22]. Conversely, as more cutting-edge therapies enter the market, costs will likely rise due to their increased complexity and genetic precision, bringing a heavy economic burden to societies, governments and healthcare systems [4]. Thus, access to treatment will become a challenging mission [4, 23, 24]. This new paradigm raises questions regarding cost-effectiveness and budget-impact issues; hence, care units must find new ways of saving resources while guaranteeing that patients receive quality care and achieve optimal results.

## Integrated care for patients with skin cancer

The primary goal of optimal care is not only motivated by mortality reduction but also to provide a better quality of life and long-term management of side effects [25]. As first addressed by Porter and Teisberg (2006) [26], a value-based healthcare (VBHC) approach aims at improving health outcomes and quality of services, measured against the cost spent by providing care to patients. Data collection and evaluation allow patients’ needs to become aligned with better and standardised treatment outcomes, reducing the impact of the burden caused by cancer [27]. Thus, healthcare provided to cancer patients will be comparable between different institutions.

**Figure 1.**
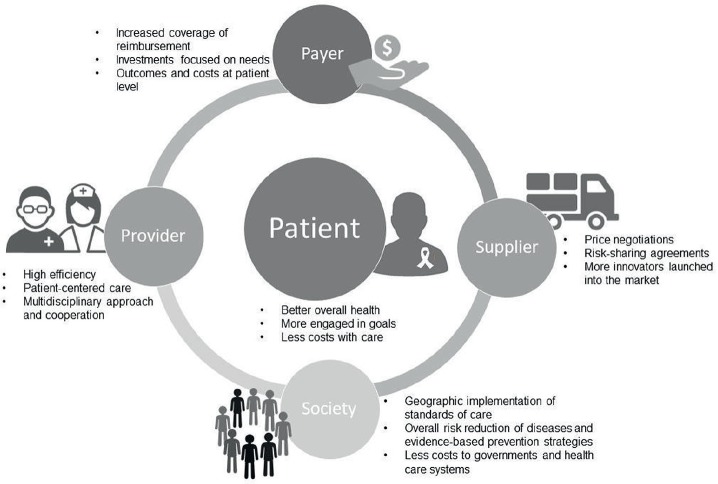
Benefits of value-based healthcare in the full-cycle system of integrated practice units to provide effective care and better survival outcomes.

Several benefits come from the introduction of a VBHC strategy to all the involved stakeholders, such as patients, healthcare providers and payers, as well as to society (Figure 1). First, VBHC models offer better health outcomes to patients, reducing the costs associated with the full-care cycle, such as those related with hospitalisations and use of medical resources [25, 27]. In these models, healthcare providers (e.g., physicians, pharmacists and nurses) are more efficient in delivering and managing patient-oriented care and more likely to engage patients in achieving the recommended goals [26]. In this way, patients could reduce attributable risk factors, through prevention and awareness campaigns, and achieve better early clinical outcomes by receiving early diagnoses and/or targeted therapies [27]. Consequently, healthcare systems are less constrained in terms of costs and the required investments are more realistic and effective, focused on specific needs.

Cancer treatments are becoming increasingly expensive together with their improved potency, complexity and innovation [27]. To allow access to those therapies at an affordable price, alternative paths of reimbursement and price regulation are being proposed, based on health technology assessment (HTA) [28]. In turn, by measuring the actual benefit/value delivered to a patient regarding treatment, it could allow fruitful price negotiations and risk-sharing agreements, for the introduction of innovators in the market [26]. Considering the VBHC approach, a shift in healthcare systems must soon occur. Current healthcare units, particularly those dedicated to cancer, must evolve to integrated, patient-centred systems, where patients are engaged in the decision-making processes and their feedback is weighted to improve cancer care and health outcomes [26, 29]. Therefore, integrated practice units (IPUs) are focused on a multidisciplinary, comprehensive, holistic plan of action that covers the entire cycle of the cancer patient’s care setting [30].

Several case studies illustrate the pathways for the creation of high-quality IPUs based on specialised, multidisciplinary care in oncology [31–34]. The retrieved evidence on the best practice pathways for patient centricity and more effective medical care in these organisations is summarised in Table 1. IPUs must be comprised of multidisciplinary teams, scalability, provision for clinical and organisational innovation, greater research and focus on outcomes measures (e.g., registries, real-world evidence and aggregated data), as well as strong notions of pharmaco-epidemiology, time and costs spending. Overall, the primary goal must be value creation for patients. As described next, to achieve this goal and develop an IPU focused on treating skin cancer, Table 2 presents practical measures for patients with melanoma, considering their different dimensions.

### Multidisciplinary team approach

A multidisciplinary-team (MDT) approach is an effective way to improve overall efficacy and to respond promptly to collateral effects [35]. This approach should include diagnostic discussion to obtain a detailed medical history, complete physical examination, laboratory and other tests, in addition to the patient’s mental and nutritional condition. During treatment, physicians can cooperate in various fields, providing more accurate monitoring conditions and insights for the treatment of melanoma (Table 2). Continuous research and development, based on partnerships with universities, pharmaceutical industries and biotechnology research centres, from national and international networks, could also generate greater knowledge exchange [23].

For those patients with special needs, such as the elderly and those with complex and multiple comorbidities, multidisciplinary meetings with other clinical specialities should be promoted to improve care management [35]. With increasing age (>70 years), other causes of death rather than cancer become more frequent, requiring the special attention of a multidisciplinary team in the detection and treatment of comorbidities. At its inception, an organisational pathway can target those patients that present the deadliest cancer, such as advanced-stage melanoma, establishing initiatives for primary prevention (change behaviours), screening and diagnosis, treatment and palliative care. Then, those procedures and models can be adapted to cover other types of cancers or other populations (e.g., geriatric patients). Overall, patient involvement is essential for an early integration of palliative care and/or other supportive care strategies.

Besides well-organised healthcare professional teams, a healthcare system based on patient-centricity must also establish better patient–physician relationships and involvement of patients in the decision-making process. Patients should be empowered and educated to participate in scientific and policy discussion, advocacy patient groups and committees as ‘patient experts’ [23, 29, 36]. In this context, patient-reported outcome measures (PROMs) are being extensively used to create the foundation for the action of this multidisciplinary approach.

### Patient-reported outcome measures

Although the ‘*one-size-fits-all*’ view is no longer applicable, it is challenging to define measures that apply to all genetic and therapeutic characteristics of each patient and bring real-life benefits to every patient [29]. Nonetheless, providing care to a selected group of patients (personalised medicine) is a way to improve results, in terms of quality, patient’s satisfaction, treatment access and efficiency [36, 37]. PROMs that capture a comprehensive perspective of the patient, considering the impact of cancer treatments on health outcomes, such as patient satisfaction, perceived quality of care, patient outcomes, symptom management and acceptability [38]. There is a trend in using the instrument EORTC QLQ30, as a complete measure across multiple domains of patient functioning (e.g., physical, role, cognitive, emotional and social), despite the low standardisation across healthcare organisations [38].

Data availability from clinical trials is still limited regarding the long-term effects of innovative targeted therapies and conventional care. Conversely, real-world evidence, based on registries and observational studies, is a useful tool to detect variability sources (e.g., differences in 1-year survival might indicate difficulties in early detection, while 5-year survival provides evidence of patient’s follow-up and treatment) [23]. Analysis of real-world data is key to evaluate patients’ outcomes, beyond the controlled environment of clinical trials, aiming at best-practice procedures (e.g., reduce the waiting time between diagnostic and start of treatment and improve the quality of services offered) [23].

Additionally, to increase the value for patients, it is essential to improve organisations of IPUs to produce comparable outcomes and establish standards of care between institutions to obtain robust and representative data [36, 37]. One possible path is to create interfaces between different health units to provide internal interactions that link steps in care and involve transferring information and/or responsibility between providers, patients and/or their respective organisations [39, 40]. These interfaces are critical to follow the entire full-cycle process, guaranteeing the continuity of patient’s care. Integrated information systems for medicine, held on e-health infrastructures and systems that give better use to available resources, should be based on the following four highlights: simplification, autonomy, monitoring and efficiency [29]. Integrated systems allow the comparison of inter- and intra-regional differences and demands, to improve sources of knowledge of clinical units and, most importantly, to measure healthcare outcomes and costs [41–43].

### Cost-effectiveness

Innovative drug therapies, especially in the advanced stages of disease, have a significant impact on cancer centres’ annual budgets [44]. Briefly, three main factors lead to cost-effectiveness and financial sustainability of an IPU focused in cancer: 1) precision medicine, 2) MDT approach and 3) patient centricity. Besides inherent differences in terms of the patients’ characteristics (e.g., age, socioeconomic status and risk factors exposure), heterogeneity exists within healthcare systems, regarding how care is offered to the patients (e.g., early diagnosis, diagnostic methods used and waiting timing between diagnosis and treatment start and type of treatments available). Differences in survival rate depend not only on the patient’s characteristics and response to treatment but also on how the treatment is delivered to patients [23, 41, 45]. Furthermore, costs depend on how the hospital resources (personnel, facilities and supplies) involved in a patient’s care process are applied and the time dedicated to the patient’s support [25, 27].

In IPUs, the patient is carefully assessed from admission through all multidisciplinary meetings, in which the case is thoroughly discussed for the best-targeted treatment approach definition. This initial evaluation allows full characterisation of patients with melanoma, from the beginning of the treatment plan, in terms of drug tolerability, the impact of other comorbidities/concomitant therapies and patient preferences. Moreover, the oversight and detailed knowledge of the subject characteristics promotes higher therapy assertiveness, reducing the risk of loss of efficacy, safety and treatment withdrawal due to patient non-adhesion or the need for therapy switch. Consequently, the IPU’s direct costs related with the management of treatment complications, such as hospitalisations and severe adverse events, are substantially reduced [25, 27]. The patient is integrated in a controlled full-care cycle, with close monitoring and involvement of different health professionals. As the internal patient flow is optimised and more effective, IPU’s resource allocation (e.g., complementary diagnosis equipment and healthcare professional time assistance) can be better employed, considering time running and avoiding unnecessary expenditures. In a more general perspective, improved clinical outcomes also reduce the burden of the disease to society, as most patients diagnosed with skin melanoma are still active workers and absence from work or even premature mortality are expected. In this context, real-world evidence allows the monitoring of clinical evidence of health technologies and provides insights about cost-effectiveness, with impact on the selection of the best-targeted care and prevention strategies to the patients [42, 43].

## Clinical, organisational and cultural features of an integrated practice unit: The Skin Clinic of IPO Porto

The Skin Clinic of *Instituto Português de Oncologia*—Porto (IPO Porto), in the North of Portugal, is a specialised IPU dedicated to different skin cancers, such as melanoma (including rare forms—uveal melanoma and mucosal melanoma) and non-melanoma cancer (e.g., Merkel Cell carcinoma, SCC and BCC). IPO Porto is a Portuguese public hospital, a national reference centre specialised in cancer treatment (11 different cancers) and member of the Organisation of European Cancer Institutes, since 2010. This organisation is also certified to the ISO 9001:2008 standard. Moreover, IPO Porto is a part of the Porto Comprehensive Cancer Centre and integrates three European Reference Networks, namely, the European Network of Cancer Registries, the European Epitranscriptomics Network and the European Network for Rare adult solid Cancer. In particular, the Skin Clinic started its activities in 2009, aiming at providing high-quality clinical treatment for skin cancer, driven by multidisciplinary care and equal healthcare delivery, to achieve the best possible patient survival and quality of life.

In 2018, IPO Porto was estimated to treat around 40,430 patients with cancer, every year, thereof 7,600 patients with skin cancers. According to the Portuguese Association of Cutaneous Cancer, it was estimated that 10,000 new cases of skin cancer are diagnosed every year in Portugal [46]. The IPO Porto treats around 26% of all melanoma cases in the country. Between 2010 and 2016, the number of new cases of malignant melanoma increased by 28%, while the incidence of non-melanoma cancer (SCC and BCC) decreased. At the Skin Clinic, patients with melanoma are mostly women (sex ratio of 1.36), of older age (> 55-year old), diagnosed with cancer at stage I/II and particularly located at lower limb (including hip), upper limb (including shoulder) and trunk. Comorbidities that may influence the medical outcomes in melanoma treatment are also identified, such as bronchitis (12.0%), dementias (4.9%) and hypertension (2.5%).

To optimise effective care and better survival outcomes, this clinic is focused on providing access to adequate healthcare services, adapting to innovation, guaranteeing enough technical and human resources and, finally, integrating new technologies/information systems. Several examples are presented in Table 3 that evidence the achievement of these objectives and the clinic’s performance in the context of a VBHC approach. As described for other IPUs of Oncology, the monitoring and analyses of these indicators allow to define internal measures and their implementation [32, 47, 48]. The creation of a patient-centred integrated healthcare system with a multidisciplinary care approach allows the mitigation of efficiency bottlenecks. Therefore, IPUs such as the Skin Clinic add value to patients by contributing to improved clinical outcomes and better quality of life, at a lower cost. Overall, this initiative involved an investment in terms of healthcare infrastructures of 581,579€, from its inception. While the introduction of innovative drug therapies became 74% of the clinic’s operational budget, the accomplished performance improvements allowed this initiative to be cost-effective within the full cycle of care. Finally, in Table 4, we describe the key metrics for accessing healthcare optimisation in the clinic throughout the full cycle.

## Conclusions

To ensure better and more cost-effective services to patients, current healthcare systems should be developed in line with institutions’ organisation and culture. Hence, the main strengths are related to standardisation of procedures and communication pathways, more involvement of clinical, non-clinical staff and patients in decision-making and more collaborations in clinical research. Currently, most healthcare institutions lack an integrated information system to measure clinical and financial outcomes.

From the perspective of personalised medicine and as newer and innovative treatments are available to patients with unmet needs, a key challenge of the current system is to understand and apply the potential of real-world evidence to support decisions in HTA. Measurement of outcomes, such as clinical, quality of life and cost, is decisive in determining affordability and access to the best available state-of-the-art care. In a multidisciplinary organisation, every health professional and non-clinical staff member should be engaged in different roles, reflecting different points of view for problem-solving. Thus, opportunities for improvement are identified by multiple team members, who discuss suggestions and ideas, seek feedback from patients, define priorities in resource needs and create effective internal communication channels and collaborations.

Among the achievements of an IPU specialised in cancer care, alignment of goals and efforts leads to improved quality, safety and efficiency of care delivery for patients and the whole organisation. With this purpose, the Skin Clinic of IPO Porto still aims at: defining standard clinical pathways for prediction of outcomes in skin cancer prevention, diagnosis, treatment and follow-up; creating a communication platform with primary care centres to exchange medical information and estimate an actual disease burden and, finally, assessing patients’ quality of life and satisfaction beyond those receiving investigational therapies. The current major limitation is that the evaluation of the quality of life was only applied for patients who used innovative and expensive treatments but is now ongoing for all the patients in the hospital. Finally, based on the actions described herein, it is possible to increase adherence to best care practices and transfer them to other institutions across the country and foresee policy recommendations that create value.

## Conflicts of interest

The author(s) declare that they have no conflicts of interest.

## Funding

The author(s) received no financial support for the research, authorship and/or publication of this article.

## Authors’ contributions

Francisco Rocha Gonçalves contributed to the conception and design of the work. Patrícia Redondo contributed to the conception and design of the work. All authors contributed to acquisition, analysis and interpretation. All authors drafted and critically revised the manuscript. All authors gave final approval and agree to be accountable for all aspects of work ensuring integrity and accuracy.

## Figures and Tables

**Table 1.** Best practice pathways and critical aspects leading to a multidisciplinary, patient-centred organisation, based on high-quality melanoma clinics.

Critical aspects	Process	Aims and achievements	Ref.
Value for patients	Identification of the key stakeholders; Definition of objectives, roles, times and areas of operation.	Standards for quality and safety of processes; Criteria for patients’ selection; Joint protocols/guidelines; Adequate facilities	[33, 34]
Continuity of care	e-health (digitalisation of medical and clinical records); Multidisciplinary teams (e.g., physician, pharmacist, nurses).	Uniformity and traceability; Data analysis and use; Collaborative networks for research projects (e.g., universities) Patient empowerment/engagement (e.g., Local Patient Advocacy Groups).	[32–34]
Outcomes measurement	Timely patient management; Cost measurement; Results-risk adjusted outcomes.	Comprehensive accreditation for high-quality oncology care; Model adaptation to other type of cancers/populations.	[31, 32]

**Table 2.** Practical measures to improve the benefit from a multidisciplinary approach in patients with skin melanoma, from diagnosis to end-stage care.

Patient dimension	Diagnosis	Treatment	Palliative/ end-stage care	Ref.
Symptoms (including psychological support)	Include general practitioners and other specialities to assess the impact of symptoms related to the disease (e.g., case discussion meetings); Structured psychosocial interventions and psychoeducation, tailored to individual experiences, should be available in clinical practice.	Use of PROMs to understand the effects of cancer therapies and areas of improvement (e.g., patients using innovative drugs report fewer side effects, such as pain, nausea and vomiting, insomnia, fatigue, dyspnoea, diarrhoea, constipation, appetite loss and anxiety).	Involvement of the patient for an early integration of palliative care and/or other supportive care strategies.	[39, 49]
Family and caregivers	Readapt the structure environment to receive the patient and his/her family (e.g., ease the scheduling of appointments, how to minimise clinical waiting times); Disseminate information (e.g., public websites, leaflets, workshops) that allows access to knowledge and exchange of experiences among the entire cancer community.	Recognise the impact and perceptions of the patients and family, during the care cycle (e.g., absences to follow-up visits); Promote trust and a clear and emphatic communication about the patient’ needs, involving caregivers and family/friends in treatment decisions.	Understand needs and manage family distress and emotional stability, at the end of life; Psychosocial support to reduce risk of psychiatric morbidity, better quality of life and less regret of patients/family and caregivers (e.g., groups meetings, supportive services).	[35, 50]
Comorbidities	Include general practitioners and other specialities to assess the impact of other comorbidities-related symptoms and differentiate from disease-specific effects.	Decide the cancer treatment recommendation based on more complete medical information about a patient, including medical history, concomitant comorbidities and therapies, and treatment preferences.	Assess the impact of comorbidities on the survival prognosis, frailty, risk of complications/life-threating complications; Offer support measures that cover also the comorbidities’ effects and associated complications.	[35, 51]
Advanced disease	Promote skin screening activities open to the community to reduce diagnoses in more advanced stages; Improve communication with geriatric and oncologist for best practice care and for assessment to determine life expectancy and treatment tolerance.	Improve understanding of the molecular and cellular changes in advanced melanoma (e.g., clinical studies in the elderly population).	Offer continuous palliative/supportive care, centralised in the unit, including specialised nurses and physical therapists.	[33, 52]

**Table 3.** Main objectives and the Skin Clinic’s performance to optimise effective care and better survival outcomes for patients with melanoma.

Key target	Objective	Performance	Value to patients
Access to innovation	Availability of proper facilities/technologies and participation in clinical trials or experimental protocols for cutting-edge therapies.	Increased 69% of differentiated reconstructive procedures and 17% number of ambulatory surgeries, after implementing two ambulatory rooms. Participation in 13 clinical trials, among which 11 referred to innovative therapies for melanoma (2012–2017).	An increase of 21% of the patients received effective treatments and patients’ quality of life and survival rate continually improved for melanoma stage I–IV.
Patient centricity	Provide multidisciplinary units, where surgeons, medical oncologists, radiation oncologists, social workers, nurses, psychologists and nonclinical professionals, intervene in the full cycle of patient’s care.	Introduction of a case manager, who evaluates, with the support of the Clinic Coordinator, the patient’s needs and uses available resources more efficiently.	Contributes to a timely and efficient schedule of exams and medical appointments, facilitating the discussion of treatment and follow-up plan in multidisciplinary consultations and, often, in group appointments within several medical specialities.
Integrated care cycle	Patient management conducted through a computerised system, for further guidance from the perspective of health technologies assessment and comparisons with external benchmarks.	Information related to the institution and the patients, including administrative and clinical data, is collected and stored in a single, centralised electronic database, from various information systems in the institution.	Data are analysed in real-time by an internal research outcomes laboratory, which measures and generates real-world evidence, namely about survival, safety, quality of life and cost of treatments.
Patient-reported outcomes (patient’s needs and quality of life)	Guarantee comfortable spaces for patients and their families, as well as access to personalised, proper and innovative diagnostic and treatment options.	The institution has also developed social networks for patients to exchange knowledge and share experiences while promoting every year, skin screening actions open to the community.	Melanoma patients reported quality of life with palliative treatment in almost 100% (best performance), for symptoms like nausea and vomiting, diarrhoea and dyspnoea.
Continuous training of healthcare professionals	Increase differentiated expertise of clinicians in specific medical conditions/pathology and promote patients’ satisfaction of healthcare and the hospital.	Participation in national/international conferences and involvement in clinical research collaborations.	Better use of medical time and recognition of patients’ priorities to maintain or recover optimal health and quality of life, including and training activities.

**Table 4.** Overall key metrics for accessing the healthcare optimisation.

Key metrics	Problematic	Measurement	Outcome
Increase access to differentiated reconstructive procedures	Improve the quality of life and satisfaction of the patients.	Number of reconstructions conducted each year, with plastic surgeons fully integrated in daily clinic activities.	Since 2010, the reconstructions procedures increased by 69%.
Increase ambulatory surgeries	Long waiting times if surgery performed in the main operating room or outpatient general rooms.	Number of ambulatory surgeries conducted in the clinic, after implementation of exclusive ambulatory rooms.	Number of ambulatory surgeries increased 17% (representing 92% of all surgeries conducted in the clinic).
Reduce waiting time for first consultation/treatment	Better use of medical times and capability of administrative staff.	Average waiting time between admission, consultation and start of treatment, after providing training to staff.	Reduced average waiting time in 20% between patient’s admission and first medical appointment, and in 39% the time between first consultations and multidisciplinary group consultations. Significantly reduced time between multidisciplinary group consultations and treatment start in 65%.
Increase patient survival	Better detection of recurrence of disease and increase patient access to innovative and more effective therapies.	Overall survival and progression-free survival of patients using innovative drugs.	Improved 5-year survival rate for melanoma patients: Stage I—100% Stage II—74.9% Stage III—55.6% Stage IV—7.6%
